# Factors Associated with E-Cigarette Use in U.S. Young Adult Never Smokers of Conventional Cigarettes: A Machine Learning Approach

**DOI:** 10.3390/ijerph17197271

**Published:** 2020-10-05

**Authors:** Nkiruka C. Atuegwu, Cheryl Oncken, Reinhard C. Laubenbacher, Mario F. Perez, Eric M. Mortensen

**Affiliations:** 1 Department of Medicine, University of Connecticut School of Medicine, Farmington, CT 06030, USA; oncken@uchc.edu (C.O.); maperez@uchc.edu (M.F.P.); mortensen@uchc.edu (E.M.M.); 2 Department of Medicine, University of Florida College of Medicine, Gainesville, FL 32610, USA; reinhard.laubenbacher@medicine.ufl.edu

**Keywords:** sole e-cigarette use, never smokers of conventional cigarettes, e-cigarette, young adults, electronic nicotine delivery system, machine learning, vaping, behavioral risk factor surveillance system, Boruta, LASSO

## Abstract

E-cigarette use is increasing among young adult never smokers of conventional cigarettes, but the awareness of the factors associated with e-cigarette use in this population is limited. The goal of this work was to use machine learning (ML) algorithms to determine the factors associated with current e-cigarette use among US young adult never cigarette smokers. Young adult (18–34 years) never cigarette smokers from the 2016 and 2017 Behavioral Risk Factor Surveillance System (BRFSS) who reported current or never e-cigarette use were used for the analysis (*n* = 79,539). Variables associated with current e-cigarette use were selected by two ML algorithms (Boruta and Least absolute shrinkage and selection operator (LASSO)). Odds ratios were calculated to determine the association between e-cigarette use and the variables selected by the ML algorithms, after adjusting for age, gender and race/ethnicity and incorporating the BRFSS complex design. The prevalence of e-cigarette use varied across states. Factors previously reported in the literature, such as age, race/ethnicity, alcohol use, depression, as well as novel factors associated with e-cigarette use, such as disabilities, obesity, history of diabetes and history of arthritis were identified. These results can be used to generate further hypotheses for research, increase public awareness and help provide targeted e-cigarette education.

## 1. Introduction

There has been a rapid increase in the use of e-cigarettes among youth and young adults in the US [1,2,3]. E-cigarettes include devices that allow users to vaporize and inhale an aerosol that typically contains nicotine, flavorings and other additives [4]. The long-term effects of e-cigarette use remain largely unknown, but e-cigarette aerosols contain toxins that can affect health [5,6,7,8]. There is increasing evidence that e-cigarettes may be associated with an increased risk of oral diseases [9,10], prediabetes [11], depression [12,13], asthma, chronic obstructive pulmonary disease (COPD) and respiratory symptoms [14,15,16,17,18]. Recently, the Centers for Disease Control and Prevention (CDC) reported multiple cases of e-cigarette or vaping product use-associated lung injury (EVALI), some of which resulted in deaths [19]. Tetrahydrocannabinol (THC)-containing e-cigarettes, or e-cigarette cartridges containing vitamin E acetate, were likely responsible for these clusters of EVALI [20]. This highlights the fact that e-cigarettes may unknowingly contain potentially harmful substances. E-cigarettes have been associated with marijuana, non-prescribed drug use and subsequent cigarette smoking, which may be explained by confounding due to common liability such as shared genetic vulnerability or environmental factors [21,22,23,24,25,26]. 

E-cigarette use is prevalent among smokers of conventional cigarettes [27], but e-cigarette use by never smokers (sole e-cigarette use) is also rising [2]. In 2016, 15% of all e-cigarette users (an estimated 1.9 million U.S. adults) were sole e-cigarette users and approximately 1.2 million of them were less than 25 years old [28]. Moreover, in 2015 and 2016, the results from two different national surveys show that 40% and 44%, respectively, of current e-cigarette users aged 18–C24 years were sole e-cigarette users [27,29]. E-cigarettes may be safer than cigarettes for smokers [30], but never smokers who use e-cigarettes likely receive little benefit [31]. Studies have shown that the perception of e-cigarettes and motivation for e-cigarette use varied based on cigarette smoking status [31,32], therefore, factors unique to never smokers need to be identified.

Young adults (18–34 years old) are more likely than older adults to report current e-cigarette use [29,33], and a significant percentage of young adults, especially 18–24year-olds, report sole e-cigarette use [2,34], but there is a paucity of research on the factors associated with e-cigarette use in this population [29]. Identifying the factors associated with e-cigarette use in young adults is critical, in light of a recent study that showed that 76% of the EVALI patients were <35 years old [35]. Additionally, knowledge of these factors is also important for regulatory authorities, because the recent FDA decision to reduce the nicotine content of combustible cigarettes may deter some individuals from initiating cigarette smoking and instead switch to the use of e-cigarettes and other noncombustible tobacco products [36]. The factors associated with e-cigarette use can be identified using machine learning (ML) techniques.

There has been an increase in the application of ML techniques to medicine and other research areas [37], but there is a paucity of the use of ML techniques in tobacco research. ML is a natural extension of traditional statistical approaches that becomes increasing valuable as the amount of data increases and the dimensionality of the dataset increases [38]. As the amount of variables to be considered increases, identifying all the variables associated with an outcome and determining the variables to be included in models becomes increasingly difficult to implement properly using standard statistical methods [38,39,40]. ML techniques can be used to identify variables associated with an outcome as the number of variables increase. ML techniques have been applied to survey data to identify variables that are associated with different psychological and disease conditions [41,42,43,44,45,46].

Variables with known relationships or exploratory guesses are used to identify factors associated with e-cigarette use. This approach may lead to the exclusion of important variables that can improve our understanding of e-cigarette use in young adults. ML techniques can reduce this limitation by automatically identifying variables associated with e-cigarette use. The goal of this study is to use ML techniques to identify demographic, behavior and health factors associated with current e-cigarette use in a representative population of young adult never smokers in the US. This is especially important because of the rapidly changing field of e-cigarette use by young adult never smokers and the potential gaps in understanding the factors associated with e-cigarette use in this population. These identified factors may be used in other models that include e-cigarette use to reduce bias due to confounding. This study will inform the work of researchers, physicians, and regulatory authorities seeking to develop programs to better target young adults at risk of sole e-cigarettes use.

## 2. Materials and Methods 

The 2016 and 2017 cross-sectional Behavioral Risk Factor Surveillance System (BRFSS) survey data were used for the analysis [47,48]. The BRFSS is a combined project between CDC and all the states in the US and participating territories. Data in the BRFSS are self-reported and collected using landlines and cellphones. The BRFSS is designed to collect data on demographics, chronic health conditions, health-related risk behaviors and the use of preventive services from the noninstitutionalized adult population (≥18 years) residing in the US and participating territories. The BRFSS includes a core set of questions that is used by all the states and optional modules that can be included by the different states. Core questions include questions about current health-related perceptions, conditions, and behaviors, as well as demographic questions. The core component includes the annual core comprising of questions asked each year to all the participants and rotating core questions that are included in even- and odd-numbered years. More information about the BRFSS design can be found elsewhere [49,50].

### 2.1. Study Population

Data from the annual core questions from the 2016 and 2017 BRFSS survey were combined as detailed in other reports [51,52] and used for the analysis. Participants were included in the analysis if they were young adults (18–34 years), were never cigarette smokers and were either current or never e-cigarette users. E-cigarette use was determined using these two questions: “Have you ever used an e-cigarette or other electronic vaping product, even just one time, in your entire life?” and “Do you now use e-cigarettes or other electronic “vaping” products every day, some days, or not at all”. Never e-cigarette users reported having never used an e-cigarette and current e-cigarette users reported currently using e-cigarettes every day or some days. Never cigarette smokers reported having smoked less than 100 cigarettes in their entire life.

There were 148,618 young adults (18–34 years). E-cigarette use and smoking status could not be ascertained for participants who reported “Don’t know/Refused/Missing” for e-cigarette use (*n* = 7585) and cigarette use (*n* = 6995). These participants were removed from the analysis. Additionally, participants who were current or former cigarette smokers (*n* = 44,418) and/or former e-cigarette users (*n* = 39,268) were removed from the analysis.

### 2.2. Data Preprocessing 

Annual core questions that were the same in 2016 and 2017 surveys were selected as variables for the analysis. Variables that were used to create other variables and variables not related to health perceptions, conditions, behaviors, or demographics (such as imputation flags, weights, and stratum) were removed from the analysis. Missing data that could be ascertained from other variables (e.g., questions that were not asked based on response to a previous question) were replaced with the appropriate categorical value. Categorical variables where participants selected “Don’t know/Not sure/Refused/Missing” were converted to a new categorical value. This was done to remove the missingness in the data [53]. Current and never e-cigarette use was combined to create a binary outcome for this analysis. After preprocessing the data, 47 variables and the outcome were selected as input for the ML algorithm.

### 2.3. Statistical Analysis Step 1: Initial Variable Selection

Boruta [54] and the least absolute shrinkage and selection operator (LASSO) [55,56] were used to select the variables that were associated with current e-cigarette use. These two algorithms will select different sets of variables, thereby reducing the likelihood of important variables being omitted. Boruta and LASSO have been used for variable selection for various types of data, such as survey, medical and genomic data [57,58,59,60,61,62,63,64].

Boruta is a wrapper built around the random forest classification algorithm. Random forest is an ensemble method where classification is performed by voting on multiple unbiased weak decision trees. Random forest can deal with nonlinear and complex relationships between the variables and the outcome. Furthermore, random forest considers the impact of each predictor variable individually, as well as in multivariate interactions with other predictor variables [65]. Boruta works by adding randomness to the data and creating randomized variables called “shadow” features. In each iteration of the algorithm, features that achieve higher importance (*Z* score) than the shadow features are counted. Variables with significantly larger importance values than the shadow variables are declared important variables, and the others are declared unimportant variables. The algorithm works to find all the relevant/important variables in the data. The important variables are those significantly correlated with the outcome. A detailed description of Boruta can be found elsewhere [54].

The LASSO algorithm puts a constraint on the sum of the absolute values of the logistic regression model parameters by applying a shrinking (regularization) process that penalizes the coefficients of the regression variables and shrinks the least important variables to zero. The tuning parameter *λ* controls the strength of the penalty. A detailed description about LASSO can be found elsewhere [55].

To avoid the errors and limitations due to a single application of a ML algorithm, and to reduce the sensitivity of the variable selection methods to small perturbations in the data [66,67], 100 iterations of Boruta and 300 iterations of LASSO with random samples consisting of 80% of the original data were performed. The features selected were stable at this number of iterations. More bootstrap iterations of LASSO were performed, because LASSO is computationally less expensive than Boruta. For LASSO, during each bootstrap iteration, a tenfold cross-validation was used to select the lambda ($\lambda_{m}$) that produced the minimum mean cross validation error [56,68]. The variables with non-zero coefficient for variables other than “Don’t know/Not sure/Refused/Missing” for $\lambda_{m}$ were selected. For both ML algorithms, the variables that were selected in ≥90% of the iterations of the bootstraps were identified as significant variables. The variables selected by either of the two algorithms were used as input to the final variable selection method.

### 2.4. Statistical Analysis Step 2: Final Variable Selection

Multivariable logistic regression was used to examine the association between e-cigarette use and the variables selected from either Boruta or LASSO, after controlling for gender, age and race/ethnicity, which are considered to be non-modifiable demographic exposures [69]. There were no statistical adjustments for the association between these non-modifiable demographic exposures and e-cigarette use [69]. Creating multivariable logistic regression models for each selected feature and adjusting for only the non-modifiable demographic exposures (gender, age, and race/ethnicity) will independently identify the factors associated with e-cigarette use. Also, in order to make the results representative of the United States noninstitutionalized young adult never smoker population, the BRFSS complex design was incorporated into the analysis, to account for the probability of selection and adjust for nonresponse bias and non-coverage errors [51,52]. The BRFSS complex data weights and analysis for the subpopulations were calculated as detailed elsewhere [70]. All analyses were conducted using R version 3.6.1, R Foundation for Statistical Computing: Vienna, Austria, 2019 [71].

Boruta package [54] was used for Boruta, glmnet package [56] was used for LASSO, and survey package [72] was used for the multivariable logistic regression. All the default parameters for Boruta were used, including mtry = square root of the number of predictor variables and ntree = 500. These are sufficient in most cases, since random forest performance has a weak dependence on its parameters [54]. *MaxRuns* was increased to 250 to prevent the algorithm from ending prematurely, thereby increasing the number of tentative features [54]. For LASSO, *cv.glmnet* in the *glmnet* package was used. *Family* was set to *binomial* and all the default parameters of *cv.glmnet* were used, including *nfold* = 10 and *alpha* = 1 [56].

## 3. Results

There were 79,539 young adult never cigarette smokers. 3,146 were current e-cigarette users and 76,393 were never e-cigarette users. Among young adult never smokers, 55.1% (95% CI 54.5–55.7) were females, 48.4% (95% CI 47.8–49.0) were white non-Hispanics, 13.7% (95% CI 13.3–14.1) were black non-Hispanics, 24.4% (95% CI 23.8–25.0) were Hispanics and 4.4% (95% CI 4.2–4.7) reported current e-cigarette use. Descriptive statistics of the variables selected by either Boruta or LASSO stratified by e-cigarette use are shown in Table 1. Variables not selected by either of the two algorithms include currently pregnant, hearing disability, a history of stroke, history of skin cancer, history of other types of cancer, history of kidney disease, history of COPD, emphysema or chronic bronchitis, history of coronary heart disease or myocardial infarction.

**Figure 1:**
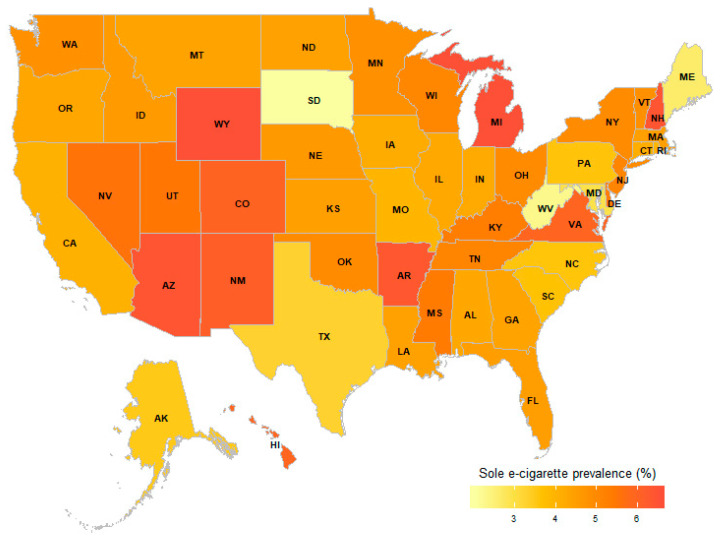
State-specific Prevalence of E-cigarette Use by Young Adult Never Smokers of Conventional Cigarettes, in 2016 and 2017 Combined.

After the initial variable selection, 38 variables were selected by Boruta and 27 variables were selected by LASSO to be significantly associated with e-cigarette use. Both algorithms selected 26 identical variables. State/territory of residence was selected by both algorithms to be significantly associated with e-cigarette use, therefore, the prevalence of sole e-cigarette use in the different states and US territories for 2016 and 2017 was calculated and shown in Figure 1 and Table S1.

Guam had the highest prevalence of sole e-cigarette use by young adults, while Puerto Rico had the lowest prevalence of sole e-cigarette use by young adults. Among the US states, sole e-cigarette use by young adults was more prevalent in Michigan and Wyoming, and less prevalent in South Dakota.

The results of the multivariable logistic regression are shown in Table 2. Three univariate logistic regressions (one for each of the following: age, gender and race/ethnicity) and 34 different multivariable logistic regressions (one for each of the selected features adjusted for age, gender and race/ethnicity) were performed. Table 2 shows the odds ratio for each selected feature after adjusting for age, gender and race/ethnicity. Variables selected by both algorithms and unique variables selected by each of the algorithms are also shown in Table 2.

Odds of e-cigarette use decreased with increasing age. Females, black non-Hispanic, other races non-Hispanic and Hispanics compared to white non-Hispanics, students compared to participants who were currently employed, and participants who had a flu shot in the past year were less likely to use e-cigarettes.

Participants who were not currently married, participants whose highest level of completed education was high school graduation compared to those who did not graduate from high school; participants who currently rent or have other arrangements, participants who could not see a doctor because of cost in the past 12 months and those who reported internet use in the past 30 days had increased odds of e-cigarette use.

Participants who were obese, who reported poor physical or mental health, who reported current smokeless tobacco use, alcohol consumption including binge drinking and heavy drinking and risky behaviors (such as occasionally driving without seatbelts, engaging in HIV risky behaviors and testing positive for HIV) had increased odds of e-cigarette use. Additionally, participants who reported vision disability, cognitive disability, independent living disability and self-care disability had increased odds of e-cigarette use. Compared with persons without the respective chronic health conditions, participants who reported a history of arthritis, diabetes, depressive disorder and participants who currently have asthma also had increased odds of e-cigarette use.

## 4. Discussion

We used an ML approach to identify previously reported as well as unreported factors associated with sole e-cigarette use in US young adults. Sole e-cigarette use differed across states. Demographic factors such as age, gender and race and other factors such as use of smokeless tobacco, alcohol consumption, engaging in risky behaviors, reporting poor mental and physical health, disabilities and chronic health conditions were associated with sole e-cigarette use.

Some of the variables selected by the algorithms have been reported previously for adult sole e-cigarette users. Mirbolouk et al. reported that adult sole e-cigarette use differed across states and the prevalence of sole e-cigarette use was highest among males and persons aged 18 to 24 years [28]. Additionally, participants who used the internet, were binge drinkers, engaged in HIV risky behaviors and reported at least 1 day with mental distress had a higher prevalence of sole e-cigarette use than non-users [28]. In another study looking at e-cigarette use in adult never smokers (never smokers included current smokers who were not smokers a year ago), black people and Hispanics had decreased odds of current and regular e-cigarette use, while unmarried participants had increased odds of current and regular e-cigarette use [73]. E-cigarette use has also been shown to be associated with alcohol use and alcohol use disorder in nonsmokers of cigarettes [74]. Associations with asthma [18] and depression [13] have also been reported for sole e-cigarette use. Thus, our ML approach agrees with the literature confirming some known factors associated with sole e-cigarette use.

Additionally, our study extends the literature on sole e-cigarette use, by identifying several new factors associated with increased odds of sole e-cigarette use. The new factors identified include vision, cognitive, self-care and independent living disabilities. Obesity, risky behaviors (driving without a seat belt and ever being tested for HIV) and chronic conditions (history of diabetes and arthritis) were also identified as associated with e-cigarette use. Additionally, home ownership and having had a flu vaccine were also identified to be associated with e-cigarette use. Further research is needed to validate these findings and to explore the nature of these associations. Some of the identified characteristics of sole e-cigarette use have been shown in cigarette smokers [75,76,77,78,79,80], which may indicate a similarity in some behavioral predictors of cigarette and sole e-cigarette use.

Most of the variables selected by LASSO were also selected by Boruta, thereby independently confirming an association between those variables and e-cigarette use. Boruta, however, selected more variables because it is a heuristic algorithm designed to find all relevant variables, including weakly relevant variables [54]. Additionally, the differences found could be due to non-linear relationships or interactions between the variables and outcomes. Some of the initial variables selected by the ML algorithms were not statistically significant after adjusting for confounders (age, gender and race/ethnicity) and the BRFSS complex design method. This may be due to the fact that the ML algorithms cannot accommodate the BRFSS complex design that adjusts for demographic differences between sampled individuals and the population they represent. Therefore, while the features were significant in the sample used for the ML algorithms, they may not have been statistically significant in the US population of never smokers. Additionally, the relationship between the selected variables and e-cigarette use may not be adequately explained by a multivariable logistic regression model. Other limitations of the ML algorithms include the fact that Boruta is computationally expensive, especially for large datasets, and LASSO has no grouping property, and as such, tends to select only one variable from a group of highly correlated variables [54,81].

Our ML approach reduces the dependence on known information and exploratory hypotheses, which are commonly used to select features that are associated with an outcome or are included in regression models. By automatically selecting features associated with an outcome, our approach reduces the possibility of missing important or previously unreported features. Furthermore, our ML approach may be used to identify features associated with an outcome as the dimension of the data increases, which is common in larger survey data. Our results show the utility of the ML approach. We were able to identify previously reported features, as well as novel features that were associated with current e-cigarette use in never smokers.

The strength of the study was the large number of participants available for the analysis, who were nationally representative of US non-institutionalized young adult never smokers. Some of the limitations include the cross-sectional nature of the analysis, the inability to establish a causality, and a lack of biochemical confirmation of e-cigarette and conventional cigarette use, which may lead to under reporting of use, which may bias the results of the analysis. Furthermore, since the data are based on self-report, there is the potential for recall bias and diagnosis misclassification bias by the participants. Our approach may have been affected by multiplicity, as we tested multiple factors associated with e-cigarette use. Additionally, the data is unbalanced, and the outcome is sparse, and this can affect the detection of some of the features associated with e-cigarette use. Moreover, the features not selected by the ML algorithms may be associated with e-cigarette use, however, those features have not been previously reported as features associated with e-cigarette use in young adult never cigarette smokers. Furthermore, we reduced the limitation of missing important features by using two different ML algorithms.

## 5. Conclusions

We were able to use machine learning algorithms to identify the factors associated with e-cigarette use in a nationally representative population of young adult never smokers. We were able to identify factors previously reported in the literature, as well as novel factors associated with e-cigarette use. Our ML approach reduces the dependence on known information and exploratory hypotheses, and reduces the possibility of missing important or previously unreported factors. Our findings may guide researchers, policy makers and health care providers, generate further hypotheses for research, increase public awareness and help provide targeted e-cigarette education on e-cigarettes use in young adult never smokers. E-cigarette products are rapidly changing, and monitoring their use patterns is a high priority for policymakers [82]. Future studies are required in order to understand the state level differences and the implications of e-cigarette use in participants with disabilities, high risk behaviors and chronic conditions.

## Figures and Tables

**Table 1:** Descriptive Statistics of the Variables Selected by Either Boruta or Least Absolute Shrinkage and Selection Operator (LASSO) for Young Adult Never Smokers Stratified by E-cigarette use.

Variables (Number = Don’t Know/Not Sure/ Refused/Missing)		Current E-cigarette User *n* = 3146 % (95% CI %)	Never E-cigarette User *n* = 76,393 % (95% CI %)
Age (mean) (*n* = 0)		22.1 (21.9–22.4)	25.8 (25.8–25.9)
Gender (*n* = 36)			
	Male	67.1 (64.4–69.8)	43.8 (43.2–44.4)
	Female	32.7 (30–35.4)	56.2 (55.5–56.8)
Race and ethnicity (*n* = 1043)			
	White only, Non-Hispanic	57.0 (54.0–59.9)	48.0 (47.4–48.7)
	Black only, Non-Hispanic	11.2 (9.2–13.1)	13.8 (13.4–14.2)
	Other race only, Non-Hispanic	8.9 (7.0–10.8)	10.5 (10.0–11.0)
	Multiracial, Non-Hispanic	2.4 (1.7–3.1)	1.5 (1.4–1.6)
	Hispanic	18.8 (16.3–21.3)	24.7 (24.1–25.3)
Marital Status (*n* = 485)			
	Married	10.1 (8.4–11.7)	31.0 (30.5–31.6)
	Not currently married ^1^	2.9 (2.0–3.8)	4.2 (3.9–4.4)
	Never married	77.0 (74.6–79.4)	56.3 (55.7–57.0)
	Member of an unmarried couple	9.5 (7.8–11.3)	7.8 (7.5–8.2)
Education level (*n* = 228)			
	Did not graduate high school	9.0 (7.3–10.7)	10.9 (10.4–11.4)
	Graduated high school	41.6 (38.7–44.5)	26.9 (26.3–27.4)
	Attended or graduated college or technical school	49.3 (46.4–52.2)	62.0 (61.3–62.6)
Employment (*n* = 888)			
	Employed for wages or self employed	58.8 (55.9–61.7)	61.2 (60.5–61.8)
	Not currently employed ^2^	11.7 (9.9–13.6)	15.8 (15.3–16.2)
	Student	28.5 (25.8–31.1)	21.9 (21.3–22.5)
Income (*n* = 13956)			
	Less than $25,000	21.9 (19.6–24.2)	24.7 (24.1–25.2)
	$25,000 to less than $50,000	20.8 (18.5–23.0)	20.0 (19.5–20.5)
	$50,000 or more	35.0 (32.2–37.8)	36.2 (35.6–36.8)
Own or rent home (*n* = 590)			
	Own a home	29.7 (26.8–32.7)	40.4 (39.8–41.1)
	Rent or other arrangements	68.4 (65.4–71.5)	58.6 (58.0–59.3)
Body Mass Index (*n* = 7328)			
	Normal weight	45.5 (42.6–48.3)	40.4 (39.8–41.0)
	Underweight	3.9 (2.8–5.1)	3.3 (3.0–3.5)
	Overweight	27.6 (25.0–30.2)	26.7 (26.2–27.3)
	Obese	19.5 (17.0–21.9)	19.6 (19.1–20.1)
Number of children in household (*n* = 460)			
	No child	61.2 (58.3–64.1)	51.6 (50.9–52.2)
	One child	20.8 (18.4–23.3)	19.3 (18.8–19.8)
	Two children	11.0 (9.2–12.9)	16.2 (15.7–16.7)
	Three or more children	6.5 (4.9–8.0)	12.3 (11.8–12.7)
Veteran (*n* = 83)		5.1 (3.9–6.2)	4.3 (4.0–4.5)
General Health (*n* = 90)			
	Good or better health	91.1 (89.6–92.5)	91.8 (91.4–92.1)
	Fair or poor health	8.9 (7.4–10.3)	8.1 (7.8–8.5)
Number of days in the past 30 days of poor physical health(*n* = 997)			
	0	61.8 (59.0–64.5)	69.4 (68.8–70.0)
	1–13	30.7 (28.1–33.3)	24.7 (24.2–25.3)
	14+	6.1 (4.9–7.3)	4.6 (4.3–4.9)
Number of days in the past 30 days of poor mental health(*n* = 866)			
	0	44.4 (41.4–47.3)	59.5 (58.9–60.2)
	1–13	36.2 (33.4–38.9)	29.8 (29.2–30.4)
	14+	18.5 (16.2–20.8)	9.5 (9.2–9.9)
Any health care coverage(*n* = 887)		82.6 (80.3–84.8)	82.6 (82.0–83.1)
Personal doctor or health care provider (*n* = 542)		60.6 (57.7–63.5)	63.1 (62.5–63.7)
Could not see doctor because of cost any time in past 12 months (*n* = 205)		14.2 (12.3–16.1)	12.9 (12.5–13.4)
Time since last routine checkup (*n* = 1696)			
	Within past 2 years	77.8 (75.3–80.4)	78.0 (77.5–78.5)
	Within past 5 years	12.0 (9.9–14.0)	10.7 (10.3–11.1)
	5 or more years ago or never	8.0 (6.3–9.7)	9.3 (8.9–9.7)
Seatbelt Use (*n* = 3059)			
	Always Wear Seat Belt	75.3 (72.7–77.8)	83.3 (82.8–83.8)
	Don’t Always Wear Seat Belt	20.6 (18.3–22.9)	12.5 (12.0–12.9)
Exercised in Past 30 Days (*n* = 1681)		82.0 (79.6–84.3)	79.1 (78.6–79.6)
Used internet in the past 30 days (*n* = 84)		98.5 (97.9–99.0)	94.7 (94.4–95.0)
Had flu vaccine in past year (*n* = 3413)		26.7 (24.1–29.2)	31.4 (30.8–32.0)
Ever had a pneumonia shot (*n* = 19,116)		28.1 (25.4–30.8)	19.6 (19.1–20.2)
Alcohol Consumption			
At least one drink in the past 30 days (*n* = 1032)		68.0 (65.1–70.8)	47.9 (47.2–48.5)
Binge drinker (*n* = 1705) ^3^		36.6 (33.9–39.4)	15.9 (15.5–16.4)
Heavy drinkers (*n* = 1908) ^4^		9.3 (7.7–10.8)	3.2 (3.0–3.4)
Currently using smokeless tobacco (*n* = 68)		7.0 (5.8–8.2)	2.1 (1.9–2.3)
Ever been tested for HIV (*n* = 5857)		32.8 (30.0–35.5)	35.4 (34.8–36.0)
HIV High Risk behavior (*n* = 4532) ^5^		23.6 (21.1–26.1)	7.4 (7.1–7.8)
Vision disability (*n* = 80) ^6^		3.5 (2.5–4.5)	2.1 (1.9–2.3)
Cognitive disability (*n* = 270) ^7^		15.8 (13.6–18.0)	7.2 (6.9–7.6)
Mobility Disability (*n* = 44) ^8^		2.5 (1.7–3.3)	2.3 (2.1–2.5)
Self-care Disability (*n* = 30) ^9^		1.6 (0.8–2.3)	0.8 (0.6–0.9)
Independent Living Disability (*n* = 96) ^10^		5.7 (4.4–7.1)	2.6 (2.4–2.8)
History of Arthritis (*n* = 241) ^11^		3.4 (2.6–4.2)	3.5 (3.3–3.7)
History of depressive disorder (*n* = 377)		20.9 (18.8–23.1)	12.1 (11.7–12.5)
History of diabetes (*n* =122)		1.8 (1.0–2.6)	1.4 (1.3–1.6)
History of Asthma (*n* = 571)			
	Currently have asthma	10.4 (8.8–12.0)	8.2 (7.9–8.6)
	No longer have asthma	8.5 (6.6–10.3)	5.5 (5.3–5.8)

^1^ Includes participants who are divorced or widowed or separated; ^2^ Includes participants who are out of work or unable to work, homemakers or retired; ^3^ Defined as ≥4 drinks for females and ≥5 drinks for males on 1 occasion in the past 30 days; ^4^ Defined as ≥7 drinks for females and ≥14 drinks for males per week; ^5^ Participant answered “yes” to whether any of the following happened in the past year: intravenous drug use, treatment for sexually transmitted or venereal disease, received money or drugs in exchange for sex, had anal sex without a condom or had four or more sex partners; ^6^ Participants answered “yes” to “Are you blind or do you have serious difficulty seeing, even when wearing glasses?”;^7^ Participants answered “yes” to “Because of a physical, mental, or emotional condition, do you have serious difficulty concentrating, remembering, or making decisions”; ^8^ Participants answered “yes” to “Do you have serious difficulty walking or climbing stairs?”; ^9^ Participants answered “yes” to “Do you have difficulty dressing or bathing?”; ^10^ Participant answered “yes” to Because of a physical, mental, or emotional condition, do you have difficulty doing errands alone such as visiting a doctor’s office or shopping?; ^11^ Participants answered “yes” to “Has a doctor, nurse, or other health professional ever told you that you had some form of arthritis, rheumatoid arthritis, gout, lupus, or fibromyalgia? (Arthritis diagnoses include: rheumatism, polymyalgia rheumatica; osteoarthritis (not osteoporosis); tendonitis, bursitis, bunion, tennis elbow; carpal tunnel syndrome, tarsal tunnel syndrome; joint infection, Reiter’s syndrome; ankylosing spondylitis; spondylosis; rotator cuff syndrome; connective tissue disease, scleroderma, polymyositis, Raynaud’s syndrome and vasculitis (giant cell arteritis, Henoch–Schonlein purpura, Wegener’s granulomatosis, polyarteritis nodosa).

**Table 2:** Association Between Current E-cigarette Use and the Variables Selected by the Machine Learning Algorithms. ^1^

	Odds Ratio (95 % CI) Boruta and LASSO ^2^	Odds Ratio (95 % CI) Boruta Only ^2^	Odds Ratio (95 % CI) LASSO Only ^2^
Gender ^3^			
Male	Reference		
Female	**0.38 (0.34–0.43)**		
Employment			
Employed for wages or self employed	Reference		
Not currently employed ^2^	0.96 (0.79–1.18)		
Student	**0.61 (0.52–0.72)**		
Race and ethnicity ^3^			
White only, Non-Hispanic	Reference		
Black only, Non-Hispanic	**0.68 (0.56–0.84)**		
Other race only, Non-Hispanic	**0.72 (0.56–0.92)**		
Multiracial, Non-Hispanic	1.33 (0.97–1.82)		
Hispanic	**0.64 (0.54–0.76)**		
Had flu vaccine in past year			
No	Reference		
Yes	**0.83 (0.72–0.95)**		
Age (mean) ^3^	**0.85 (0.84–0.86)**		
Own or rent home			
Own a home	Reference		
Rent or other arrangements	**1.23 (1.05–1.43)**		
General Health			
Good or Better Health		Reference	
Fair or Poor Health		**1.26 (1.03–1.53)**	
Body Mass Index			
Normal weight		Reference	
Underweight		0.92 (0.67–1.27)	
Overweight		1.12 (0.97–1.30)	
Obese		**1.29 (1.08–1.55)**	
History of Asthma			
No			Reference
Currently have asthma			**1.33 (1.11–1.61)**
No longer have asthma			1.26 (0.98–1.62)
Number of days in the past 30 days of poor physical health			
0	Reference		
1–13	**1.33 (1.17–1.52)**		
14+	**1.79 (1.41–2.26)**		
History of Arthritis			
No		Reference	
Yes		**1.39 (1.06–1.81)**	
Education level			
Did not graduate High School	Reference		
Graduated High School	**1.46 (1.15–1.85)**		
Attended or graduated College or Technical School	1.14 (0.90–1.45)		
Could not see doctor because of cost any time in past 12 months			
No	Reference		
Yes	**1.52 (1.28–1.81)**		
Seatbelt Use			
Always Wear Seat Belt	Reference		
Don’t Always Wear Seat Belt	**1.52 (1.30–1.77)**		
Number of days in the past 30 days of poor mental health			
0	Reference		
1–13	**1.53 (1.33–1.75)**		
14+	**2.49 (2.08–2.99)**		
Marital Status			
Married	Reference		
Not currently married	**2.46 (1.69–3.57)**		
Never married	**1.60 (1.27–2.02)**		
Member of an unmarried couple	**2.27 (1.72–2.98)**		
Ever been tested for HIV			
No	Reference		
Yes	**1.75 (1.52–2.02)**		
Visual disability			
No		Reference	
Yes		**1.76 (1.27–2.45)**	
History of diabetes			
No		Reference	
Yes		**1.86 (1.16–2.96)**	
History of depressive disorder			
No	Reference		
Yes	**2.12 (1.84–2.44)**		
Cognitive disability			
No	Reference		
Yes	**2.33 (1.94–2.81)**		
Independent living disability			
No		Reference	
Yes		**2.42 (1.82–3.31)**	
Used internet in the past 30 days			
No	Reference		
Yes	**2.48 (1.70–3.63)**		
Self-care disability			
No		Reference	
Yes		**2.60 (1.50–4.52)**	
Currently using smokeless tobacco			
No	Reference		
Yes	**2.69 (2.18–3.32)**		
Binge drinker			
No	Reference		
Yes	**3.56 (3.12–4.06)**		
At least one drink in the past 30 days			
No	Reference		
Yes	**3.64 (3.14–4.21)**		
Heavy drinkers			
No	Reference		
Yes	**3.67 (3.01–4.48)**		
HIV High Risk behavior			
No	Reference		
Yes	**3.68 (3.16–4.29)**		
Number of children in household			
No child		Reference	
One child		1.03 (0.88–1.21)	
Two children		0.90 (0.73–1.10)	
Three or more children		0.79 (0.60–1.05)	
Length of time since last routine checkup			
Within past 2 years	Reference		
Within past 5 years	1.11 (0.90–1.36)		
5 or more years ago or never	0.94 (0.65–1.35)		
Has personal doctor or health care provider			
No		Reference	
Yes		0.95 (0.83–1.08)	
Has any health care coverage			
No		Reference	
Yes		0.99 (0.83–1.18)	
Exercised in Past 30 Days			
No		Reference	
Yes		1.01 (0.86–1.20)	
Income			
Less than $25,000	Reference		
$25,000 to less than $50,000	1.14 (0.94–1.37)		
$50,000 or more	1.10 (0.92–1.30)		
Veteran			
No		Reference	
Yes		1.13 (0.88–1.45)	
Ever had a pneumonia shot			
No	Reference		
Yes	1.15 (0.99–1.33)		
Mobility disability			
No	Reference		
Yes	1.38 (0.96–1.99)		

^1^ May be affected by multiplicity as we tested multiple factors associated with e-cigarette use; ^2^ Adjusted for age, sex and race/ethnicity. Bolded odds ratios are statisitically significant; ^3^ Univariate logistic regressions. Not adjusted by age, sex and race/ethnicity.

## Supplementary Materials

The following are available online at https://www.mdpi.com/1660-4601/17/19/7271/s1. Table S1: State and US territory-specific prevalence of current e-cigarette use among young adults who are never-smokers of conventional cigarettes.
