# Supplementary material for: Factors Associated with E-Cigarette Use in U.S. Young Adult Never Smokers of Conventional Cigarettes: A Machine Learning Approach
Source: Int J Environ Res Public Health. 2020 Oct 5;17(19):7271. doi: 10.3390/ijerph17197271 (PMC7579019; doi:10.3390/ijerph17197271)
Supplement: Supplementary file 1 [file ijerph-17-07271-s001.pdf]

Table S1: State and US territory-specific prevalence of current e-cigarette use among young adults who are never-smokers of conventional cigarettes

State / Territory	Current Sole E-cigarette User % (95% CI %)	State / Territory	Current Sole E-Cigarette User % (95% CI %)
Guam	8.7 (5.4–11.9)	Massachusetts	4.5 (2.8–6.1)
Michigan	6.5 (5.1–8.0)	Kansas	4.5 (3.4–5.5)
Wyoming	6.5 (4.0–9.0)	Montana	4.4 (2.6–6.1)
New Hampshire	6.4 (3.3–9.4)	North Dakota	4.4 (2.8–6.0)
Arizona	6.4 (4.9–7.9)	Louisiana	4.4 (2.6–6.3)
Arkansas	6.3 (3.1–9.5)	Georgia	4.4 (3.0–5.8)
New Mexico	6.1 (4.0–8.2)	Alabama	4.3 (2.7–5.8)
Colorado	6.0 (4.7–7.3)	Oregon	4.3 (3.0–5.6)
Hawaii	5.9 (4.4–7.4)	Illinois	4.3 (2.8–5.7)
Virginia	5.9 (4.4–7.4)	Indiana	4.2 (3.1–5.4)
Nevada	5.6 (3.4–7.7)	Iowa	4.2 (3.0–5.4)
Utah	5.5 (4.5–6.5)	Connecticut	4.1 (2.8–5.5)
Mississippi	5.4 (3.2–7.5)	California	4.0 (3.1–5.0)
Kentucky	5.3 (3.3–7.2)	Missouri	3.9 (2.4–5.4)
Delaware	5.3 (3.1–7.5)	Rhode Island	3.9 (2.0–5.7)
New Jersey	5.1 (3.5–6.7)	South Carolina	3.6 (2.5–4.8)
Tennessee	5.1 (3.1–7.0)	North Carolina	3.6 (2.1–5.0)
New York	5.0 (3.9–6 .0)	Pennsylvania	3.6 (2.4–4.8)
Wisconsin	5.0 (3.3–6.8)	Alaska	3.5 (1.0–5.9)
Ohio	4.9 (3.6–6.3)	Texas	3.3 (2.1–4.5)
Oklahoma	4.9 (3.1–6.8)	Maryland	2.9 (1.9–3.8)
Vermont	4.8 (2.7–7.0)	Maine	2.6 (1.4–3.8)
Washington	4.8 (3.8–5.8)	District of Columbia	2.5 (1.2–3.8)
Minnesota	4.8 (3.9–5.7)	West Virginia	2.2 (1.1–3.4)
Idaho	4.6 (2.9–6.2)	South Dakota	2.0 (0.8–3.3)
Nebraska	4.6 (3.3–5.9)	Puerto Rico	0.8 (0.3–1.4)
Florida	4.5 (3.4–5.6)		
